# 915. Hepatitis A Burden of Disease and Associated Complications in Children and Adults in Mexico: A Retrospective Database Study from 2000-2019

**DOI:** 10.1093/ofid/ofab466.1110

**Published:** 2021-12-04

**Authors:** Gerardo Luna-Casas, Gilberto Sanchez-Gonzalez, Maria Yolanda Cervantes Apolinar, Gloria Huerta, Adriana Guzman Holst

**Affiliations:** 1 Estimatio SC, Mexico City, Distrito Federal, Mexico; 2 Instituto Nacional de Salud Pública (INSP), Mexico City, Distrito Federal, Mexico; 3 GSK, Mexico, Mexico city, Distrito Federal, Mexico; 4 GSK, Panama, Panama City, Cocle, Panama

## Abstract

**Background:**

In Mexico, Hepatitis A virus (HAV) infection is the leading cause of viral hepatitis in children, yet the pediatric HAV vaccine is not included in the national immunization program (NIP). Since 2013, 1-dose HAV vaccine is only given to infants of agricultural workers and in day-care centers. Mexico has intermediate HAV endemicity, yet the real burden of symptomatic HAV is unknown. The objective of this study is to describe the burden of HAV infection and severe complications in children and adults in Mexico.

**Methods:**

A retrospective database study was performed using the national surveillance system from all public health institutions in Mexico. Data on laboratory/clinically confirmed HAV cases, hospitalizations, and deaths, including for severe complications (Fulminant Hepatic Failure, Acute Liver Failure, Liver transplant), from 2000 to 2019 were analyzed (**Table 1**). Descriptive analyses were performed to estimate the disease burden and direct medical costs due to HAV per year for all ages, sex, and region.

Table 1. Definition HAV-associated disease based on ICD-10 codes

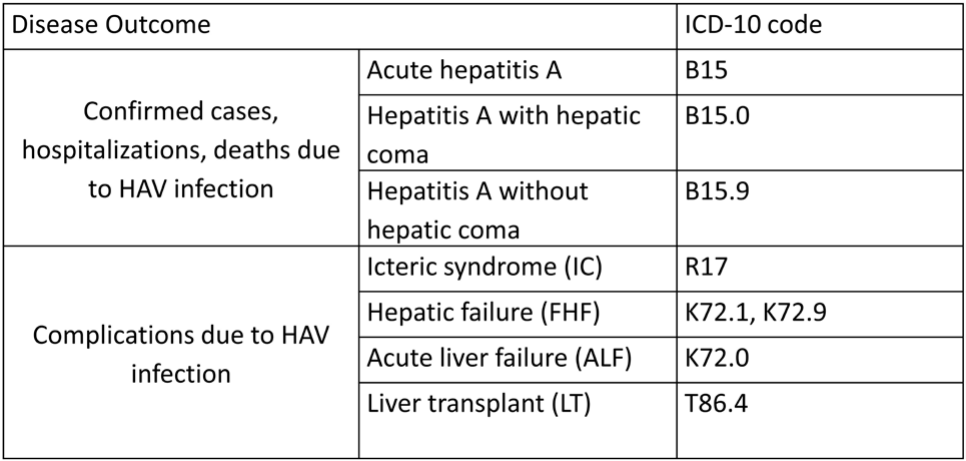

HAV, Hepatitis A Virus; ICD-10, International Classification of Diseases 10 Classification System

**Results:**

During the analysis period, the average annual incidence was 29.4 per 100,000 population (range: 43.0–10.9); the average hospitalization rate/year was 5.8% (range: 2.9%– 9.6%); and the average fatality rate/year was 0.44% (range: 0.23%–0.83%) (Figures 1a-b). Overall, there was a decreasing trend in HAV incidence over 2000–2017, with a recent increase in 2017–2019. As the incidence risk of HAV infection decreased, the mean age of infection increased. The biggest burden of HAV continued to be in children (1–9 years-old), yet there was an increase in incidence and hospitalizations (with complications) in older age groups (≥ 10–64 years-old) (Figures 2-3). The total direct medical costs (2019) due to HAV and related complications was estimated at &3.8 million Mexican pesos, which represented about 0.063% of total 2019 expenditure of the federal public budget for the health function.

Figure 1. (a) Hospitalization rate (%) due to HAV infection by year in Mexico. (b) Fatality rate (%) due to HAV infection by year in Mexico. HAV, Hepatitis A Virus

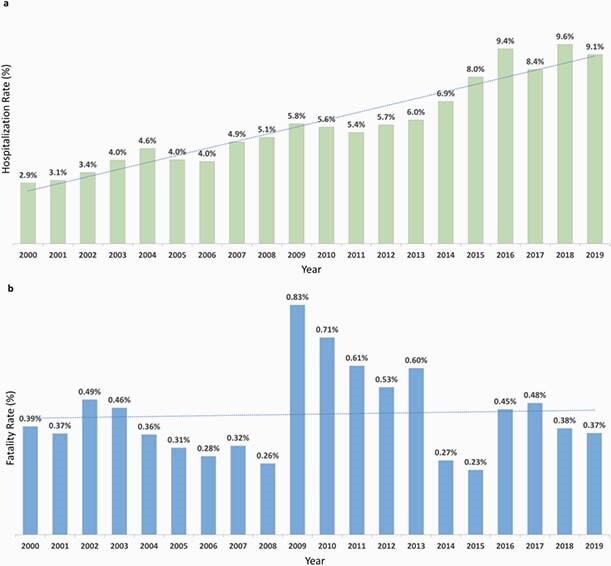

Figure 2. Incidence of HAV cases by age group and year in Mexico. HAV, Hepatitis A Virus; YoA, Years of Age

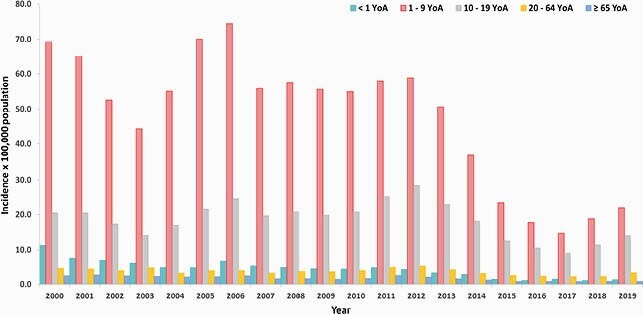

Figure 3. Hospitalizations due to HAV infection and complications by age group and year in Mexico. HAV, Hepatitis A Virus; YoA, Years of Age

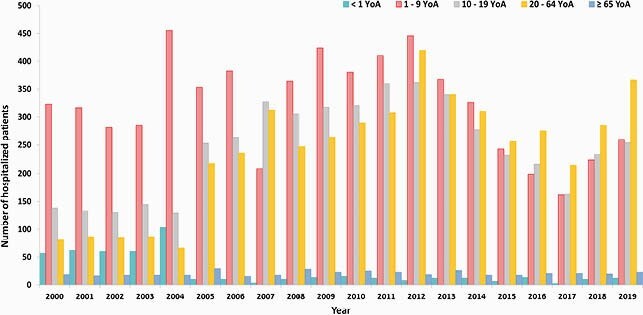

**Conclusion:**

In addition to Mexico’s intermediate HAV endemicity and improved sanitary conditions, these results describe an increase of HAV infection from children to adolescents/adults, which increases the risk for more severe and complicated disease and greater demand on healthcare resources. Our findings support the evidence for HAV vaccine inclusion in the NIP in Mexico.

**Disclosures:**

**Gerardo Luna-Casas, MD**, **The GSK Group of Companies** (Scientific Research Study Investigator, Other Financial or Material Support, Company Owner (Estimatio SC)) **Gilberto Sanchez-Gonzalez, PhD**, **The GSK Group of Companies** (Scientific Research Study Investigator, Other Financial or Material Support, Company Owner (Estimatio SC)) **Maria Yolanda Cervantes Apolinar, MD**, **The GSK Group of Companies** (Employee, Shareholder) **Gloria Huerta, MD**, **The GSK Group of Companies** (Employee, Shareholder) **Adriana Guzman Holst, MPH**, **The GSK Group of Companies** (Employee)

